# Response to Bridging the Gaps in Dementia Care: A Call for Integrated Comorbidity Management

**DOI:** 10.1002/hsr2.70832

**Published:** 2025-05-15

**Authors:** Yuchen Zhang, Zhirui Deng, Jennifer B. Seaman, Theresa A. Koleck

**Affiliations:** ^1^ School of Nursing University of Pittsburgh Pittsburgh Pennsylvania. USA

Dear Editor,

We thank the commentator for their letter to the Editor entitled “Bridging the Gaps in Dementia Care: A Call for Integrated Comorbidity Management” in recognition of our recent publication that utilized the *All of Us Research Program* data set and comprehensively examined the patterns and prevalence of comorbidities in people living with dementia [1]. We appreciate the perspectives shared in this letter, which acknowledged the importance of our research objectives and findings in the original publication about comorbidities in dementia care [2].

In this letter, the author highlighted the significance of considering health outcomes along with the paradigm shift of addressing the overall disease profile and well‐being of people living with dementia [1]. In addition to the health outcomes mentioned in the commentary (i.e., hospitalization rates, functional decline, and mortality), our research team is examining symptom burden including behavioral, neuropsychiatric, and distressing symptoms, which is another important and underexplored dimension of care for people living with dementia. Given the neurodegenerative nature of dementia and the gradual decline in communicative abilities, the presence of distressing symptoms can easily be overlooked or undermanaged, leading to compromised quality of life and psychological well‐being in both patients and their caregivers [3, 4, 5, 6]. Drawing insights from symptom science in oncology research, we aim to develop symptom clusters based on patients' overall disease profiles and relevant biomarkers to help unravel the complexity of dementia care [7]. This line of investigation further generates care‐related knowledge that enables patients and their families to develop tailored plans based on their unique situations while helping healthcare clinicians proactively prepare for individualized care scenarios and disease‐related decision‐making.

The author from the commentary also reflected importance of an interdisciplinary approach in facilitating comprehensive care management in people living with dementia requiring an interdisciplinary approach while ensuring adequate access to resources [1]. Currently, the *Guiding an Improved Dementia Experience (GUIDE) Model* provides meaningful initial steps in establishing infrastructures for comprehensive dementia care, such as supplying 24/7 support services in managing both dementia‐related syndromes and co‐occurring comorbidities and facilitating communications among primary care and specialty healthcare providers [8]. Future development should take into account of factors related to socioeconomic status and rural‐urban differences in accessing health‐related services and resources as well [9].

Although limitations exist in using electronic health records, ongoing investigations can improve the robustness of data validity by refining computable phenotypes for identification of individuals living with dementia and determination of diagnosis with comorbidities, such as incorporating medications, laboratory results, and self‐reported health outcomes in additional to diagnosis codes [10]. In combination with longitudinal analysis, we will not only gain a better understanding of the progression of the overall disease profile in people living with dementia but also further develop tailored dementia care management plans centered around each individual's needs and values [9, 11].

## Author Contributions

**Yuchen Zhang:** conceptualization, writing – original draft, writing – review and editing. **Zhirui Deng:** writing – review and editing, conceptualization. **Jennifer B. Seaman:** conceptualization, writing – review and editing. **Theresa A. Koleck:** conceptualization, writing – review and editing.

## Conflicts of Interest

The authors declare no conflicts of interest.

## Transparency Statement

The corresponding author (Yuchen Zhang) affirms that this manuscript is an honest, accurate, and transparent account of the study being reported; that no important aspects of the study have been omitted; and that any discrepancies from the study as planned and study registration is not applicable.

## Data Availability

The authors have nothing to report.
